# A proposal for the future of scientific publishing in the life sciences

**DOI:** 10.1371/journal.pbio.3000116

**Published:** 2019-02-12

**Authors:** Bodo M. Stern, Erin K. O’Shea

**Affiliations:** Howard Hughes Medical Institute, Chevy Chase, Maryland, United States of America

## Abstract

Science advances through rich, scholarly discussion. More than ever before, digital tools allow us to take that dialogue online. To chart a new future for open publishing, we must consider alternatives to the core features of the legacy print publishing system, such as an access paywall and editorial selection before publication. Although journals have their strengths, the traditional approach of selecting articles before publication (“curate first, publish second”) forces a focus on “getting into the right journals,” which can delay dissemination of scientific work, create opportunity costs for pushing science forward, and promote undesirable behaviors among scientists and the institutions that evaluate them. We believe that a “publish first, curate second” approach with the following features would be a strong alternative: authors decide when and what to publish; peer review reports are published, either anonymously or with attribution; and curation occurs after publication, incorporating community feedback and expert judgment to select articles for target audiences and to evaluate whether scientific work has stood the test of time. These proposed changes could optimize publishing practices for the digital age, emphasizing transparency, peer-mediated improvement, and post-publication appraisal of scientific articles.

## Abstract 2

This Perspective article proposes new practices for scientific publishing that align better with today's digital environment than do legacy practices.

## Introduction

### An outdated publishing process that is costly and delays access to knowledge

Most scientific work in the life sciences is still disseminated using a process inaugurated by the Royal Society in the 17th century, with the notable addition of peer review in the middle of the 20th century. This process starts with authors submitting a manuscript to the journal of their choice, where editorial selection and peer review culminate in an editorial thumbs-up or thumbs-down decision that determines whether the article is accepted for publication or rejected. If it is rejected, sometimes after several rounds of peer review, the author starts all over again at a different journal, typically until the paper gets accepted for publication somewhere.

When print was expensive, it made sense for journals to select what to publish and to charge subscription fees for a hard copy of the journal. But the internet allows us to envision and build a more open publishing environment in which nobody is shut out of research results through subscription paywalls, and internet- and community-driven approaches—not editorial selection before publication—filter the literature. The traditional journal approach of selecting papers before publication strikes us as outdated because it is often slow, costly, and harmful for science. It is slow and costly for authors and funders, because cycles of rejection and revision consume time and resources that may improve articles but also create an opportunity cost for advancing science in new directions. The delay in getting research published hurts science because research that isn’t published is equivalent to research not done, at least from the perspective of the broader scientific community and the public. Editorial selection also consumes the time and resources of selective journal publishers and peer reviewers, who spend much of their effort rejecting papers. A high rejection rate makes it expensive for selective journals to switch to an open-access publishing model because open-access fees are currently collected for published, not rejected, articles. Editorial selection before publication therefore raises the cost of open-access publishing and impedes the full transition to open access. Finally and most importantly, editorial selection before publication is harmful for science because it has turned scientific publishing into a game of “getting into the right journals,” which shapes academic career advancement and the behavior of scientists in undesirable ways.

### The publishing game and how it shapes behavior and evaluation of scientists

Despite the costs, scientists play this game of “getting into the right journals” because winning it has become crucial for advancing a scientific career. Funding, hiring, and promotion decisions are increasingly influenced by where scientists publish, not what they publish [1]. Alarmingly, the next generation of scientists already anticipates needing to publish in certain journals to be competitive for faculty positions. The bibliometric indicator that is most widely used in the evaluation of scientists is the journal impact factor (JIF)—the average citations in a given year garnered by all articles published in the journal over the two previous years. Much has been said about why the JIF is a poor quality indicator for individual articles [1]. For example, two journals may have largely similar distributions of article citations but markedly different JIFs because of outliers, such as heavily cited papers [2–4].

Despite these shortcomings, funders and employers continue to use the JIF in funding and employment decisions. The JIF rose to prominence during the expansion of the life science community, because funding and hiring panels began increasingly confronting two assessment challenges—large numbers of candidate scientists and specialized research areas outside the expertise represented by the panels. The solution that all decisions should be made by experts “who read the papers” does not easily scale to situations in which evaluators lack either time or expertise to evaluate the work’s intrinsic merit. Therefore, if we want to dissuade from the use of journal-level metrics in the evaluation of scientists, we will need to develop mechanisms that make expert evaluations more discoverable, including easy-to-use article-level metrics that reflect relevant quality features of individual articles.

Although journals shouldn’t be blamed for an academic incentive and reward system that uses their selections as a shorthand in academic evaluations, they clearly contribute to the persistent use of journal-level metrics. Journals have become brands that promote their name (and impact factor) to the scientific community. Journal branding stifles discoverable and article-level evaluations of scientific work in the following ways:

- Most journals keep peer reviews confidential among editors, reviewers, and authors. This secrecy gives editors more flexibility to decide what to publish, but it leaves the community with the publishing decision as the only visible outcome of the peer review process and thus the journal brand and the JIF as the only evident indicators of quality and significance.
- High–impact-factor journals maintain position at the top of the journal hierarchy by keeping selectivity artificially high. High–impact-factor journals in the life sciences are publishing fewer papers today than three decades ago, despite significant growth of the life sciences community [5]. Just like shoppers willing to pay a high price for a Gucci bag, scientists are willing to spend more effort on publishing in high–impact-factor journals to benefit from the recognition that comes with these publications. These efforts in turn undermine scientists’ motivation to participate in alternative evaluation schemes that could replace journal-level with article-level metrics.
- Journal branding conflicts with the correction of publication errors. Although journals retract papers with serious flaws, most erroneous publishing decisions are not corrected by journals. They are discounted among experts, whereas the flawed, misinterpreted, or overinterpreted articles continue to appeal to unsuspecting funding and hiring panels.

The forces of nontransparent quality controls, artificial selectivity, and articles “written in stone” all contribute to the persistence of the JIF and to prestige journal brands. Getting into these journals can, for some scientists, even become more important than actually getting the science right. In this hypercompetitive publishing environment, researchers are tempted to exaggerate their work’s impact, to choose research topics deemed suitable for top journals, and to refrain from open sharing of data and other research outputs.

In summary, although the traditional journal-based publishing process has strengths, it too often restricts access through paywalls, wastes resources, delays dissemination of research findings, and shapes the evaluation and behavior of scientists in undesirable ways.

## Recommendations

To drive scientific publishing forward, we propose several long-term changes. Although these changes could be implemented independently, together they promise to significantly increase transparency and efficiency.

1. Change peer review to better recognize its scholarly contribution.
2. Shift the publishing decision from editors to authors.
3. Shift curation from before to after publication.

### 1. Change peer review to better recognize its scholarly contribution

We could increase the quality of peer review and provide recognition for this activity [6–8] if we made it:

#### Transparent

Publishing peer review reports and author responses for a manuscript, anonymously or with attribution, would reveal the rigor of the peer review process and open up to interested readers the scholarly exchange that accompanies the publication of an article. Today, transparency is the exception. We believe it should become the norm.

#### Journal-agnostic

Peer review that is independent of journals focuses on feedback to the authors and evaluation of technical quality and originality of the submitted work. It reverses the trend in which peer review has morphed into a means of assisting editors in deciding whether a paper is suitable for their journal. It is important to identify papers of unusual significance and broad interest, and peer reviewers can contribute to that appraisal in a journal-agnostic fashion by properly describing the originality and scientific context of the work in question.

#### Consultative

Consultations among peer reviewers—whether through virtual feedback on individually submitted peer reviews or a joint report after consultation among reviewers [9]—could effectively eliminate unreasonable reviewer demands.

If peer review becomes a more constructive dialogue through these measures, reviewers may increasingly opt to sign their reviews. Signing of peer reviews ultimately aligns better with the notion that peer review deserves credit as a labor-intensive scholarly activity and important service to the scientific community. But we recognize that peer reviewers may not be as forthcoming with their critiques if signing reviews becomes compulsory, particularly when the author is an established scientist who may be able to affect the career of the reviewer. The technology exists to allow peer reviewers to remain anonymous while still receiving credit for their peer review efforts. This “dynamic anonymity” protects vulnerable reviewers but gives them the option to lift their anonymity at a later time. One step toward recognizing peer reviewers would be to index peer reviews with their own digital object identifier (DOI), as some journals and Publons already do [10,11], making it possible to cite peer reviews and include them on the peer reviewer’s curriculum vitae (CV) or open researcher and contributor ID (ORCID) profile.

### 2. Shift the publishing decision from editors to authors

The independence of scientists is at the heart of the research enterprise. Indeed, academic scientists lead the design and the execution of their own research plans after obtaining a principal investigator position and funding. This concept that scientists are in charge of the research process should be extended to the final step of the research workflow—the dissemination of the primary research results. Today, journal editors decide when primary research is published. Shifting the publishing decision from editors to authors would fundamentally change the roles and motivations of authors, peer reviewers, and editors and open the door to publishing and evaluation practices that, we believe, are right for the digital age.

Authors would benefit from deciding when to publish original and revised articles because they could avoid excessive rounds of peer review and revisions they consider unnecessary. Reviewers would benefit because their peer review burden would decrease. But this shift begs the question of how authors can be motivated to only publish rigorous work and not prematurely rush to publication. The following are a few considerations.

- The scientific community and publishers should define appropriate standards and quality controls that must be satisified by all author-posted articles (including checks for plagiarism, data and/or image manipulation, author affiliations, data availability, violation of the law, etc.). PLOS and bioRxiv are collaborating on certification badges that signal quality controls that have been perfomed on author-posted preprints [12]. Open data and materials badges, developed by the Center for Open Science, are used at several journals [13,14]. In some research areas, including public health and clinical medicine, it may be best to filter out or correct ill-designed research earlier in the research workflow through preregistration of the research plans [15].
- Publishing the peer review reports increases visibility of quality control, keeps authors honest, and motivates constructive feedback.
- A desire to have one’s articles selected and highlighted by editors and peers will continue to motivate authors to publish high quality work. In an author-driven publishing process, article selection (curation) would happen after publication (see below).

We believe it makes sense that, in an open publishing environment, research is shared by authors and then scrutinized through discoverable peer review and filtering mechanisms after publication. If discoverable, peer review reports and post-publication evaluations contribute to the authors’ reputation and become powerful motivators for authors to publish their best possible work and revise it in response to reasonable and legitimate reviewer concerns. And if an author decides to stand by a paper despite serious criticism from peers, at least those criticisms will be accessible to readers, who can decide for themselves whether they agree with the author’s or the reviewers’ point of view. This is arguably better than the situation today, in which authors can publish any work somewhere, though not necessarily in their journal of choice, usually without critical reviews that highlight potential shortcomings.

### 3. Shift curation from before to after publication

How could scientists find work of interest in a sea of primary articles posted by authors and improved by peer reviewers? This requires filtering or curation of primary research articles for specific audiences (e.g., experts) or broader audiences. Experts would find valuable content in their own research field through powerful search engines. Because search algorithms will only get better in the future, we expect that many specialized journals that currently curate a large fraction of the literature will become obsolete.

Time-consuming curation by experts would be more critical for research findings that claim to be highly significant and of broad interest, because many potential readers may not have the expertise or time to evaluate the work themselves. In the current publishing ecosystem, selective journals serve this important curation function by selecting papers for publication. Given that scientists continue to rely on these selections, we can presume these journals do as good a job as one can expect curating articles prior to publication. But the accuracy of these selections would improve after publication, when the scientific community has time to interact with the work and start the process of validation. Pre-publication curation is, in effect, a bet on the future influence of a selected article, whereas post-publication curation is closer to reporting wins or losses. In particular, post-publication curation offers the following advantages.

- Post-publication curation can leverage the community of expert users—scientists who actually use, reproduce, and build on the published data. Peer review before publication has become a serious challenge because life sciences research is increasingly interdisciplinary with data analysis, not data generation, as the rate-limiting step. How can we expect the few peer reviewers to verify significance and conceptual advances before publication, when this job could take a significant fraction of the time it took the authors to produce their analysis in the first place, and when the reviewer’s expertise often doesn’t cover all aspects of the described work [16]?
- Post-publication curation can continue over time and highlight many different features of articles, unlike a one-time, thumbs-up publishing decision at journals.
- Post-publication curation can in principle cover the entire published literature, beyond articles submitted to a particular journal.
- Effective post-publication curation offers the promise of alternatives to journal-based metrics like the JIF.

## Implementation

### Publishing platforms

Author-driven dissemination in the life sciences already exists on publishing platforms. The defining feature of publishing platforms is that they empower authors to make publishing decisions. Preprint servers share author-posted articles before they undergo peer review at a journal, with no delay to dissemination. The arXiv preprint server has been used for decades in the physics community. Preprints enjoy increasing popularity in the life sciences, with bioRxiv as the biggest server [17]. Publishing platforms powered by F1000Research infrastructure go further: authors publish preprints, orchestrate the peer review process for that preprint, and publish the revised article [18].

Open-access publishing platforms are positioned to increasingly complement—and perhaps eventually replace—journals as major publication venues for primary research articles. High-volume publishing on these platforms allows primary research to be published faster, because authors decide when to publish original and revised articles. The platforms are typically cheaper than journals because they don’t include expensive editorial curation associated with high rejection rates. Overall, the combined cost of publication on platforms and post-publication curation can be significantly lower than the current cost of journals because many primary research articles may not need post-publication curation; in fact, some fraction of specialized articles may not even need formal peer review, because the few scientists who access these articles on preprint servers can quickly evaluate rigor and quality of the data themselves.

We envision a platform infrastructure that enables different providers to offer diverse services—publication of versioned articles from preprints to the final version of record, quality controls before publication, peer review, copy editing, post-publication curation, etc. One business model would enable service providers to charge a fee for service. Competition among service providers could create an environment of experimentation on publishing platforms that would, over time, identify the most valuable and cost-effective services. There is an argument for research funders to financially support publishing platforms and the services that run on them, at least until publishing volume has increased to levels that can sustain the platforms through service fees. Current leaders in this effort include the Wellcome Trust and the Bill and Melinda Gates Foundation, which support their own open research platforms [19,20], and the Chan Zuckerberg Initiative, which supports bioRxiv [21].

Some journals could become publishing platforms over time, shedding editorial gatekeeper roles. A publishing trial at *eLife* is exploring the impact of forgoing editorial rejection after peer review [22]. The editorial gatekeeper role before peer review could be replaced when it becomes feasible and culturally acceptable to use community approaches or algorithms to allocate peer reviewer resources wisely. The F1000Research platform uses authors instead of editors to select reviewers [18]. Bioverlay uses academic editors to select preprints for peer review. Alternatively, scientists could self-select to review preprints [23]. Further experimentation with different peer review models could improve efficiency and quality of peer review on publishing platforms.

### Curation journals

As high-volume publishing platforms continue to grow, we’ll need curation services that select articles of interest for specific target audiences. Today’s selective journals and scientific societies could be well positioned to provide such services. Future curation journals could retain many of their current features, including subscription income, independent editors and editorial boards, and nontransparent evaluations. They could exploit the above-mentioned advantages over the curation at traditional “publishing” journals in the following ways.

#### Post-publication curation could be multidimensional, with articles selected based on different criteria

Some selection criteria could be similar to those journals use today, including “of broad interest,” “of unusual significance,” “potentially groundbreaking but controversial,” or “rigorous and elegant.” Alternatively, curators could flag articles that are personal favorites, as F1000Prime does. A particularly valuable curation service would be to identify significant claims in published articles that are questionable or could not be validated in the community. Today, such judgments are largely restricted to the privacy of expert circles. If discoverable through reputable curation services, these judgments could motivate authors to publish rigorous research. The multidimensional nature of curation after publication means that it could capture nuances and complexities much better than traditional journals, in which both “positive” and “negative” curation typically boil down to simple decisions—whether to publish or whether to retract.

#### Post-publication curation should take full advantage of the internet and community input

To illustrate what this input could look like, consider the following process: Every few months, an advisory board of experts nominates articles for a given set of categories. The selection that follows the nomination process can be informed by a mix of crowdsourcing, i.e., tallying the votes of board members, and editorial judgment, i.e., weighing comments from board members. The combination of community input and independent editorial oversight ensures that the selection process is not a simple popularity contest. Selection of an article is signaled by tagging the article with a badge (see below) and can be justified with a short review.

Post-publication curation journals confront at least two significant challenges.

#### Peer review burden

Established scientists are already overburdened with peer review requests from journals. On the upside, scientists wouldn’t be asked to conduct a full technical peer review but to select articles they (and their trainees) have already dissected in detail. And starting post-publication curation with early-career group leaders and trainees may further mitigate this challenge: early-career scientists are not yet overburdened with peer review duties, have the most to gain from building an alternative evaluation and reward system in academia, and are still directly involved in hands-on research. Trainees could be engaged in curation through mentored journal clubs, similar to their engagement in the review of preprints through preprint journal clubs [24]. Ultimately, if publishing on platforms becomes mainstream and post-publication curation is recognized as a critical service for science, the burden on scientists and editors will shift from deciding what to publish to curating what is already published.

#### Business model

Even if curation after publication is deemed highly valuable, it remains an untested business model. Research funders could support curation services initially, but academic libraries would ultimately have to subscribe to them to make them sustainable. It may be difficult to monetize the selection outcome itself, but scholarly reviews that justify the selections could be subscription worthy. There are opportunities to develop and experiment with new models of curation to explore what is most valuable for the scientific enterprise and sustainable for providers.

### Alternatives to the JIF

One way to dissuade the use of journal-level metrics like the JIF in the evaluation of scientists is to develop better proxies that reflect quality features of articles. Post-publication curation offers an opportunity to develop such proxies in the form of “badges.” Badges would capture the selection of articles in a shorthand form and would be attached to papers in a discoverable and searchable manner. Like post-publication curation, badges could be multidimensional. Authors could contribute to badging through structured citations—e.g. by flagging research and methods articles that were foundational for a given article, similar to the practice at the Current Opinions journals, which mark references that are “of outstanding interest” and “of special interest.” (For more ideas on making citations more useful, see [25,26].) Curation journals could contribute to badging by signaling that the selected paper satisfies the journal’s editorial standards, conveying the same information as current publishing decisions. Other badges could be generated directly on publishing platforms by aggregating peer reviewer scores or by including short summary statements that distill key aspects of the paper—originality, significance, key findings, remaining reviewer concerns, target audience, etc.

Finally, some badges could take full advantage of internet capabilities and be generated automatically through crowdsourcing and analytics over time (citations, readability, data usage, etc.). Altmetrics represent one existing example of article-specific metrics that aggregate citations, downloads, social media mentions, etc. [27]. A disadvantage of usage and citation metrics is that they are lagging indicators that take a long time to acrrue. To compete with a leading indicator like the JIF, it would be important to start post-publication curation and badging soon after publication while still taking advantage of community input. Because curation and badges are not “written in stone” like a publishing decision, they can be revised over time, providing a mechanism for modifying or correcting earlier judgments.

### Outlook

We envision a publishing model in which dissemination and curation of scientific work are separated, making both processes more efficient and effective [28,29]. To achieve a transition to open publishing platforms and post-publication curation, the scientific community needs the self-awareness and courage to make a significant cultural shift.

Successful scientists have a vested interest in the current system. The notion that publication itself serves as a trusted “stamp of quality” is deeply ingrained, and scientists adhere instinctively to a more or less agreed-upon hierarchy of journals for judgment, reading preferences, and evaluations. As authors, they chose journals based on prestige and quality but are shielded from associated publishing costs because their libraries pay for subscription licenses. As peer reviewers, they provide critical and time-consuming services and, in exchange, gain privileged access both to unpublished work and to the “right” editors. Scientists may also interpret a call for change as an implicit criticism that they don’t execute their role well enough despite investing significant effort. It is easy to blame individuals, like editors or the “third reviewer,” for mistakes in publishing decisions. But the problems we describe here are systemic and not resolved by tackling individual misjudgments that will always be part of scientific evaluations.

Over time, however, we believe that the scientific community will come to support a progressive open publishing model that accelerates discovery and empowers scientists. Authors would spend less time and resources on getting their work published, and peer reviewers might need to review less often. Even post-publication curation could turn out to be effort neutral if it grows at the expense of pre-publication curation. To move forward, we encourage the community to push for progress on core issues such as the following.

How can we optimize the structure of peer review and the selection of peer reviewers for platform publishing? How do we determine what level of peer review an article needs in the first place—none, basic, premium? How can the peer review reports be structured—with scores and short statements of key features—to contribute most effectively to subsequent post-publication curation and badges?

How do we set up an infrastructure and culture for post-publication curation? How do we decide on suitable categories for selection? How should we identify work that didn’t stand the test of time?

Finally, what business models are best suited to support sharing of primary research articles on platforms and post-publication curation?

Publishers, scientific societies, academic institutions and their libraries, and funders can play critical roles in addressing these issues. Publishers can experiment with publishing platforms. Scientific societies can use the expertise of their members to orchestrate fee-for-service peer review on publishing platforms and subscription-based curation services. Libraries may be able to support curation journals when publication of primary research articles shifts towards cheaper publishing platforms, liberating funds that are currently spent on traditional subscription journals.

Research funders are uniquely positioned to promote change because we sit at the nexus of two interconnected functions—the sharing of research outputs and the evaluation of scientists. Many funders see it as our responsibility to support practices that disseminate research outputs openly and efficiently and evaluate scientists’ work based on intrinsic merit. The evaluation of scientists in academia places heavy emphasis on where and how much they publish, rather than what they publish. If these incentives don’t change, scientists will continue to publish in a manner that perpetuates the current problems. Changes in academic incentives cannot come from publishers. Funders and academic institutions need to commit to evaluate science and scientists independent of the publication venue [1]. Developing and sharing principles on how to evaluate scientists and learning from each other how to implement them will set us on a path to better incentives and rewards for rigorous and enduring research. One example of work in this area is the Open Research Funders Group, a community of practice.

In addition to supporting changes in the academic incentive system, funders can catalyze changes in publishing by encouraging and supporting publishing platforms, pilot studies on peer review, and new forms of post-publication curation. Such pilots should measure their impact on authors, reviewers, and readers and should be scalable. Their outputs should contribute to the evaluation of scientists and scientific work.

By fostering an environment for experiments in publication and evaluation and continuously assessing and building on effective practices, we can together develop services that best support science in the digital age. We stand to gain fairer, more effective ways to communicate findings, share data, and develop the next generation of scientists. At Howard Hughes Medical Institute, we believe this is the future of publishing. We are moving toward it.

## Abbreviations

CV: curriculum vitae
DOI: digital object identifier
HHMI: Howard Hughes Medical Institute
JIF: journal impact factor
ORCID: open researcher and contributor ID
