# Correction: A proposal for the future of scientific publishing in the life sciences

**DOI:** 10.1371/journal.pbio.3000179

**Published:** 2019-03-06

**Authors:** The PLOS Biology Staff

A section below the Acknowledgements thanking the reviewers for their contributions was inadvertently removed from the manuscript during the production process. The section in full can be found below, the publisher apologizes for the error.

## Reviewers

The three peer reviewers of this manuscript—Richard Sever, Judd Walson and Bjoern Brembs—revealed their identities and agreed to be listed as such in the manuscript. We thank them for their contribution to the process.
